# Factors Associated With Endowed Chair Allocation in Medical Oncology in the United States

**DOI:** 10.1093/jncics/pkac036

**Published:** 2022-05-12

**Authors:** Lena Jia, Michael Kevin Rooney, Clare E Jacobson, Kent A Griffith, Christina Mangurian, Reshma Jagsi, Merry Jennifer Markham

**Affiliations:** Washington University School of Medicine, St Louis, MO, USA; Department of Radiation Oncology, The University of Texas MD Anderson Cancer Center, Houston, TX, USA; The University of Michigan Medical School, Ann Arbor, MI, USA; Center for Bioethics and Social Sciences in Medicine, University of Michigan Medical School, Ann Arbor, MI, USA; Department of Psychiatry and Behavioral Sciences, University of California, San Francisco, CA, USA; Department of Radiation Oncology and The Center for Bioethics and Social Sciences in Medicine, University of Michigan Medical School, Ann Arbor, MI, USA; Division of Hematology and Oncology, University of Florida, Gainesville, FL, USA

## Abstract

To explore persisting gender disparities across leadership roles in medicine, we examined factors associated with holding endowed chairs in US oncology divisions. In 2019, we identified 95 academic oncology divisions, using the Oncology Division Chiefs and Department Chairs listing in the American Society of Clinical Oncology myConnection forum. We collected public information on gender, degree, total National Institutes of Health funding as principal investigator, H-indices, publication and citation numbers, and graduation year and constructed a multivariable logistic regression model. All statistical tests were 2-sided. We identified 1087 oncology full professors. Of these, 287 (26.4%) held endowed chairs: 60 of 269 women (22.3%) vs 227 of 818 men (27.8%) (*P* = .08). On multivariable analysis, greater research productivity and National Institutes of Health funding were associated with having an endowed chair (*P* < .001), whereas gender was not (*P* = .45). Though sample size was limited, if gender differences are in fact smaller in certain subspecialties than other fields of internal medicine, insights might emerge to guide efforts to promote equity.

Despite an increasing number of female physicians in the workforce, gender disparities remain across leadership roles throughout the field of medicine (1,2). Endowed chairs are recognized by their institutions as having achieved excellence in their field. Because this prestige provides a plethora of research and career opportunities, endowed chairs are coveted in academia (3). We examined factors associated with holding endowed chairs in oncology divisions across the United States, with a focus on whether a gender difference exists, as has been demonstrated in top internal medicine departments (4).

In 2019, we identified 95 academic oncology divisions or departments in the United States using the Oncology Division Chiefs and Department Chairs listing in the American Society of Clinical Oncology myConnection forum. We requested a list of full professors and endowed chairs in those divisions or departments and relied on available information on an institution’s official website when an institution did not respond. Using public data (eg, institutional websites, National Institutes of Health [NIH] RePORTER, Scopus, state licensing boards), we collected information on gender (attributed by investigators into binary categories), degree, total NIH funding as principal investigator, H-indices, publication and citation numbers, and year of graduation from terminal degree for these individuals. We then created a multivariable logistic regression model to examine if, after controlling for other variables, gender was independently associated with an increased likelihood of holding an endowed chair. The research plan was filed with the University of Michigan Institutional Review Board, which did not consider it to require regulation. Two-sided *P* values were calculated using the Wald test; *P* values less than .05 were considered statistically significant.

A total of 1087 oncology full professors were identified, of whom 269 were women. Overall, 287 (26.4%) held endowed chairs: 60 of 269 women (22.3%) and 227 of 818 men (27.8%) (Table 1). Overall, and in an adjusted model, greater research productivity (as measured through publications, citations, and H-index) and greater levels of NIH funding were statistically significantly associated with having an endowed chair (*P* < .001 for all). Gender was not statistically significantly associated with endowed chair status (22.3%, 95% confidence interval [CI] = 17.4% to 27.8% of female professors and 27.8% [95% CI = 24.7% to 31.0%] of male professors held endowed chairs; *P* = .08) on bivariable analysis, nor was it statistically significant in the adjusted multivariable model (*P* = .45; odds ratio = 0.87, 95% CI = 0.61 to 1.24, for female vs male) (Figure 1).

**Figure 1.**
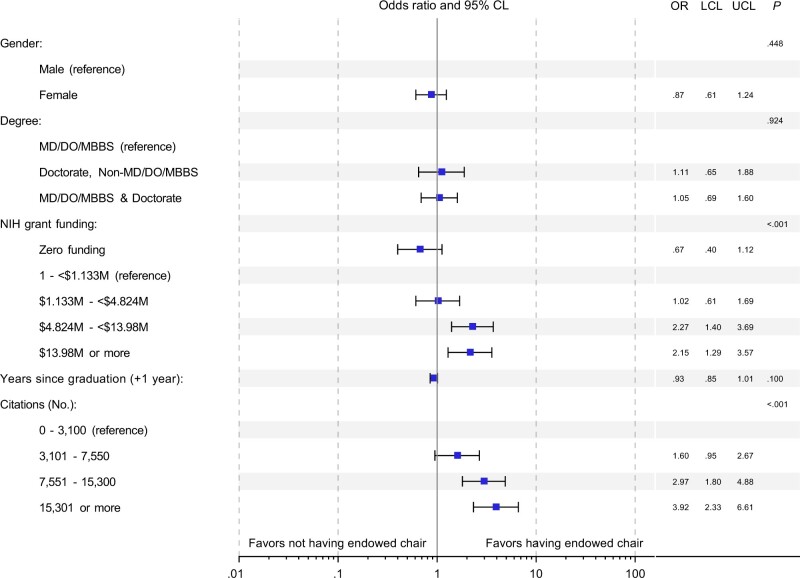
Multivariable logistic regression model explaining having an endowed chair for full professors. Funding data were initially categorized into those with funding and those without; individuals with any funding were further subcategorized by quartile for multivariable analysis. Citation data were categorized by quartile. Publication and H-index data were excluded from the multivariable model due to collinearity with citations. CL = confidence limit; LCL = lower confidence limit (fifth percentile); OR = odds ratio; UCL = upper confidence limit (95th percentile). *P* values (reported in the last column) are 2-sided, Wald-based *P* values for the logistic odds ratio estimates from the multivariable model, which test whether the odds ratio is statistically significantly different from 1 for each covariate or indicator; *P* values less than .05 were considered statistically significant.

**Table 1.** Factors associated with endowed chair allocation among full oncology professors, with results displayed separately by gender

Characteristic	Women (n = 269)		Men (n = 818)	
	With endowed chairs	Without endowed chairs	With endowed chairs	Without endowed chairs
	(n = 60)	(n = 209)	(n = 227)	(n = 591)
	No. (%)	No. (%)	No. (%)	No. (%)
Degree				
MD or equivalent	45 (21.1)	168 (78.9)	161 (25.7)	466 (74.3)
MD + other doctorate	10 (34.5)	19 (65.5)	38 (32.8)	78 (67.2)
Doctorate, non-MD or equivalent	5 (18.5)	22 (81.5)	26 (35.6)	47 (64.4)
Other	0 (0)	0 (0)	2 (100)	0 (0)
NIH grant funding level as PI^a^				
No funding	9 (10.3)	78 (89.7)	28 (13.5)	179 (86.5)
<$1 133 000	8 (16.7)	40 (83.3)	29 (19.7)	118 (80.3)
$1 133 000 to <$4 824 000	11 (22.4)	38 (77.6)	31 (21.4)	114 (78.6)
$4 824 000 to <$13 980 000	20 (39.2)	31 (60.8)	56 (39.4)	86 (60.6)
$13 980 000+	11 (34.4)	21 (65.6)	80 (48.2)	86 (51.8)
Not applicable/none reported	1 (50)	1 (50)	3 (27.3)	8 (72.7)
Total publications^a^				
Not available	0 (0)	1 (100)	0 (0)	4 (100)
0-75	12 (13.3)	78 (86.7)	26 (13.8)	162 (86.2)
76-135	14 (19.4)	58 (80.6)	41 (20.4)	160 (79.6)
136-230	20 (32.3)	42 (67.7)	54 (29.0)	132 (71.0)
231+	14 (31.8)	30 (68.2)	106 (44.4)	133 (55.6)
Total citations^a^				
Not available	0 (0)	2 (100)	0 (0)	4 (100)
0-3100	11 (12.5)	77 (87.5)	21 (11.4)	164 (88.6)
3101-7550	12 (17.9)	55 (82.1)	41 (20.4)	160 (79.6)
7551-15 300	19 (30.2)	44 (69.8)	63 (31.5)	137 (68.5)
15 301+	18 (36.7)	31 (63.3)	102 (44.7)	126 (55.3)
H-Index^a^				
Not available	0 (0)	1 (100)	0 (0)	4 (100)
0-27	11 (12.5)	77 (87.5)	21 (11.1)	169 (88.9)
28-41	17 (23.6)	55 (76.4)	42 (21.6)	152 (78.4)
42-58	15 (25.4)	44 (74.6)	64 (30.6)	145 (69.4)
59+	17 (34.7)	32 (65.3)	100 (45.2)	121 (54.8)
Decade of graduation from terminal degree				
Not reported	0 (0)	3 (100)	6 (27.3)	16 (72.7)
1960 or earlier	1 (100)	0 (0)	3 (27.3)	8 (72.7)
1961-1970	1 (25.0)	3 (75.0)	13 (24.5)	40 (75.5)
1971-1980	14 (29.8)	33 (70.2)	56 (24.5)	164 (74.6)
1981-1990	24 (21.4)	88 (78.6)	83 (28.1)	212 (71.9)
1991-2000	18 (20.0)	72 (80.0)	63 (31.7)	136 (68.3)
2001 or later	2 (16.7)	10 (83.3)	3 (16.7)	15 (83.3)

a Funding data were initially categorized into those with funding and those without; individuals with any funding were further subcategorized by quartile for multivariable analysis. Citation, publication, and H-index data were categorized by quartile. NIH = National Institutes of Health; PI = principal investigator.

Given the sample size, post hoc power analyses suggested that the minimally detectable statistically significant difference in endowed professorship proportions by gender was approximately 8% with standard power of 80% and 5% type 1 error. Power to detect the observed difference given the total number of professors and their gender distribution was found to be approximately 45%, suggesting a 55% chance of failing to reject the null hypothesis of equal gender distributions in endowed chairs when the observed difference is as large as estimated.

Within this sample of oncology full professors, gender was not statistically significantly associated with endowed chair status, although the number of full professors in this field is too small to definitively rule out a modest difference. This finding contrasts with prior work that revealed a substantial difference by gender that remained statistically significant after controlling for similar variables in a study examining all divisions in the departments of internal medicine at top medical institutions (1). Although the differences in these results may reflect insufficient power to detect a small gender-based difference in endowed chair allocation in the present study, it is also important to consider the possibility that there may be true differences in the study populations. The observation in this sample that women constituted such a small share, less than 25%, of the full professors suggests that even if differences in subsequent success in attaining endowed chairs do not exist after individuals reach the rank of full professor, efforts to ensure equal opportunities to achieve the rank of full professorship in this field remain necessary, because women have constituted more than 40% of medical oncology and hematology-oncology fellows in the United States since 2002 (5). If gender differences in leadership and recognition are smaller in certain specialties compared with other fields of internal medicine, insights might emerge to guide efforts to promote equity, diversity, and inclusion in other fields. This study adds to the existing literature by demonstrating that not all subspecialties of internal medicine necessarily have the same gender disparities, and research of this sort in other fields would provide a more complete understanding to guide interventions.

Limitations of this study include sample size, the use of publicly available data to determine demographic characteristics, and the binary definition of gender. We were also unable to account for differences in how endowed chairs are selected at different institutions. Further work is necessary to understand what specialty-specific and institutional cultural factors may contribute to professional inequity and whether oncology or other specialties of medicine might exemplify policies or practices that other fields might adopt. Such work will be instrumental for informing targeted action to aid in the pursuit of diversity, equity, and inclusion (6).

## Funding

No funding was used for this study.

## Notes

**Role of the funder:** Not applicable.

**Disclosures:** Dr Jagsi has stock options as compensation for her advisory board role in Equity Quotient, a company that evaluates culture in health care companies; she has received personal fees from the Greenwall Foundation, Doris Duke Foundation, and the National Institutes of Health and grants for unrelated work from the National Institutes of Health, the Doris Duke Foundation, the Greenwall Foundation, the Komen Foundation, and Blue Cross Blue Shield of Michigan for the Michigan Radiation Oncology Quality Consortium. She has a contract to conduct an investigator-initiated study with Genentech. She has served as an expert witness for Sherinian and Hasso, Dressman Benzinger LaVelle, and Kleinbard LLC. No other disclosures were reported.

**Author contributions:** LJ: Data curation, Investigation, Writing—original draft, Writing—review and editing. MKR: Data curation, Investigation, Writing—original draft, Writing—review and editing CEJ: Data curation, Investigation, Writing—review and editing. KAG: Data curation, Formal Analysis, Methodology, Validation, Visualization, Writing—review and editing. CM: Conceptualization, Methodology, Writing—review and editing. RJ: Conceptualization, Methodology, Project administration, Resources, Supervision, Writing—original draft, Writing—review and editing. MJM: Conceptualization, Investigation, Methodology, Project administration, Supervision, Writing—review and editing.

## Data Availability

The data underlying this article will be shared on reasonable request to the corresponding author.
